# Bacterial and viral fecal indicator predictive modeling at three Great Lakes recreational beach sites

**DOI:** 10.1016/j.watres.2022.118970

**Published:** 2022-08-10

**Authors:** Mike Cyterski, Orin C. Shanks, Pauline Wanjugi, Brian McMinn, Asja Korajkic, Kevin Oshima, Rich Haugland

**Affiliations:** a U.S. Environmental Protection Agency, Office of Research and Development, Athens, GA, 30605, United States; b U.S. Environmental Protection Agency, Office of Research and Development, Cincinnati, OH 45268, United States; c New York State Department of Health, Center for Environmental Health, Bureau of Water Supply Protection, New York City Watershed Section, Albany, NY 12201, United States

**Keywords:** Great Lakes, Coliphage, qPCR, Fecal indicators, Statistical modeling

## Abstract

Coliphage are viruses that infect *Escherichia coli* (*E. coli*) and may indicate the presence of enteric viral pathogens in recreational waters. There is an increasing interest in using these viruses for water quality monitoring and forecasting; however, the ability to use statistical models to predict the concentrations of coliphage, as often done for cultured fecal indicator bacteria (FIB) such as enterococci and *E. coli*, has not been widely assessed. The same can be said for FIB genetic markers measured using quantitative polymerase chain reaction (qPCR) methods. Here we institute least-angle regression (LARS) modeling of previously published concentrations of cultured FIB (*E. coli*, enterococci) and coliphage (F+, somatic), along with newly reported genetic concentrations measured via qPCR for *E. coli*, enterococci, and general *Bacteroidales*. We develop site-specific models from measures taken at three beach sites on the Great Lakes (Grant Park, South Milwaukee, WI; Edgewater Beach, Cleveland, OH; Washington Park, Michigan City, IN) to investigate the efficacy of a statistical predictive modeling approach. Microbial indicator concentrations were measured in composite water samples collected five days per week over a beach season (~15 weeks). Model predictive performance (cross-validated standardized root mean squared error of prediction [SRMSEP] and $R_{\text{PRED}\;}^{2}$) were examined for seven microbial indicators (using log_10_ concentrations) and water/beach parameters collected concurrently with water samples. Highest predictive performance was seen for qPCR-based enterococci and *Bacteroidales* models, with F+ coliphage consistently yielding poor performing models. Influential covariates varied by microbial indicator and site. Antecedent rainfall, bird abundance, wave height, and wind speed/direction were most influential across all models. Findings suggest that some fecal indicators may be more suitable for water quality forecasting than others at Great Lakes beaches.

## 1. Introduction

Statistical models have seen increased use for predicting water quality in recreational waters in recent years (Christensen et al., 2021; Francy et al., 2020; Jones et al., 2013). This trend is partly due to the realization that persistence models (using water quality measures taken one day to predict quality on the following day) are often inaccurate (Whitman and Nevers, 2008), and waiting 8–24 h for cultured microorganisms to be counted (USEPA, 2021) reduces the level of public health protection in recreational waters (Wymer et al., 2021). Statistical predictive models could allow for public advisories to be issued quickly enough to inform beach attendance on the same day of use (Brooks et al., 2013).

Recreational water quality fecal indicator data, such as cultured enterococci and *E. coli,* are often modeled using least squares fitting via a multiple linear regression framework (de Brauwere et al., 2014). Least-angle regression with Lasso (LARS-lasso, Efron et al. (2004)) modifies the linear regression approach by constraining the sum of absolute regression coefficients (i.e., L_1_ regularization), providing a method for identifying important/unnecessary covariates, filtering highly collinear covariates, and reducing the overfitting of training data. Linear regression-based methods, although sometimes outperformed by machine learning techniques like random forests and boosted models (Brooks et al., 2016; Thoe et al., 2012), may be less susceptible to overfitting with smaller datasets (*n <* 100) that are typically available for recreational water quality forecast modeling.

Although cultured fecal indicator bacteria (FIB) predictive modeling is well established, research suggests that viruses are a more likely causative agent of many recreational waterborne illnesses compared to bacterial pathogens (Begier et al., 2008; Sinclair et al., 2009; Soller et al., 2010). As a result, scientists are investigating the use of coliphage as an alternative fecal indicator for water quality testing. Coliphage (F+ and somatic) are viruses that infect coliform bacteria, including *E. coli*, and may be effective indicators of the presence of human fecal contamination and the associated risk from enteric viruses because they are consistently found in municipal sewage and are similar in size and structure to some human enteric viruses (Havelaar et al., 1993; King et al., 2011; McMinn et al., 2017a). Coliphage are found in the digestive systems of humans and other warm-blooded animals (McMinn et al. 2017a) and are routinely identified in sewage (Ewert and Paynter, 1980; Gantzer et al., 1998; Korajkic et al., 2020; Lucena et al., 2004). Coliphage are also accepted metrics for microbial monitoring of groundwater sources of drinking water (USEPA, 2006).

In addition, there is a growing interest in using genetic methods for recreational beach monitoring. The primary advantage of these methods over culture-based protocols is the ability to provide water quality information within a few hours (Griffith and Weisberg, 2011). Several epidemiological studies report a significant relationship between the incidence of swimming-related illnesses and genetic enterococci concentration estimates determined by quantitative polymerase chain reaction (qPCR) methods in both marine and Great Lakes recreational waters (Colford et al., 2012; Wade et al., 2008; Wade et al., 2010). Based on these findings, the United States Environmental Protection Agency (EPA) has suggested Beach Action Values associated with qPCR-based EPA Method 1611 for enterococci (USEPA, 2012a, 2012b). A health relationship has also been demonstrated for qPCR estimates of total *Bacteroidales* by EPA Method B (USEPA, 2010c) in marine waters (Wade et al., 2010).

This study implements LARS-lasso models and uses cross-validation to compare the predictive performance of previously-reported (Wanjugi et al., 2018) coliphage (F+ and somatic), cultured FIB (*E. coli* and enterococci), and newly-presented (this study) qPCR-based genetic marker (*E. coli*, enterococci and *Bacteroidales*) concentrations for three Great Lakes sites across a single recreational beach season (15-weeks). Covariates include previously reported (Wanjugi et al., 2018) paired measurements of common water and beach area parameters routinely used to describe and model water quality conditions in recreational settings ranging from water temperature to rainfall (Francy, 2009; Francy et al., 2013; USEPA, 2010a, 2010b). Emphasis is placed on the comparative predictive performance of each fecal indicator response variable utilizing a standardized modeling approach. In addition, the most influential covariates are identified to reveal potential trends that could inform future recreational water quality sample testing and predictive modeling efforts. Findings suggest that some fecal indicators may be more suitable for water quality forecasting than others at Great Lakes beaches.

## 2. Materials and methods

### 2.1. Site descriptions

**Fig. 1.**
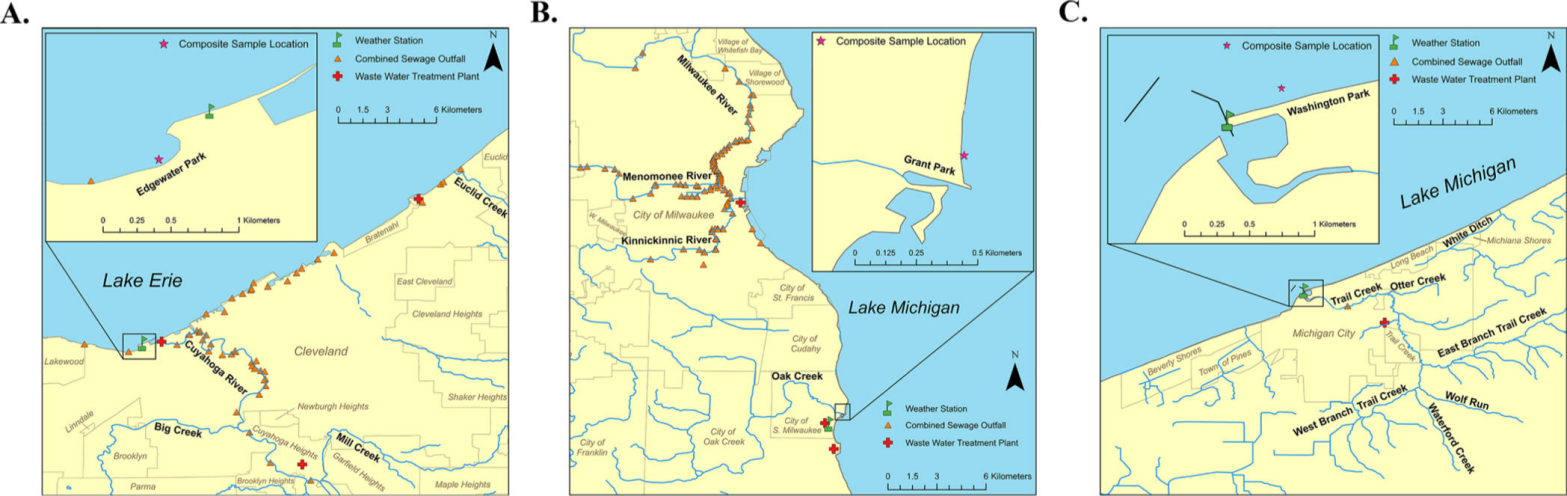
Three Great Lakes beaches sampled for microbial water quality: Edgewater Beach in Cleveland, OH (A), Grant Park in South Milwaukee, WI (B), and Washington Park in Michigan City, IN (C). Modified from Wanjugi et al. (2018).

Sites included Edgewater Beach near Cleveland, OH, Grant Park in South Milwaukee, WI, and Washington Park in Michigan City, IN (Fig. 1, modified from Wanjugi et al., 2018). All are routinely monitored and have yielded FIB concentrations that exceed the USEPA’s recommended Beach Action Values in 10–30% (USEPA, 2012b) of samples based on historical monitoring. At these sites, potential fecal pollution sources are a mixture of nearby wastewater treatment facilities, stormwater runoff, tributary inflows, and combined sewer overflows, with secondary influence from beachgoers, wildlife, and agricultural runoff. For additional details, see Wanjugi et al. (2018).

### 2.2. Water sampling

Water samples for microbial indicator testing were collected from late May to early September of 2015. Each sampling event produced a 6 L composite created from six 1 L grab samples collected in a transect area (three shin-deep and three waist-deep). As described in Wanjugi et al. (2018), water samples were collected via standard methods recommended in Section 9060 of Standard Methods for the Examination of Water and Wastewater (APHA, 2005). Samples were collected at approximately 8:30am on each sampling day (Monday through Friday). The total number of sampling events was 71 at Grant Park, 67 at Washington Park, and 67 at Edgewater Beach.

### 2.3. Cultured bacteria and viral fecal indicator datasets

This study uses previously-published data on the concentrations of cultured FIB (enterococci and *E. coli*) and coliphage (F+ and somatic) from Wanjugi et al. (2018). Briefly, cultured *E. coli* counts (most probable number, MPN/100ml) were obtained using Colilert Quantitray (Idexx, Westbrook, ME). Cultured enterococci concentrations (colony forming units, CFU/100ml) were determined by membrane filtration on mEI agar (USEPA, 2009). A dead-end hollow fiber ultrafiltration with single agar layer (D-HFUF-SAL) method was used to enumerate F+ and somatic coliphage (plaque forming units, PFU/L) as described in McMinn et al. (2017b) and the Supplementary Materials of Wanjugi et al. (2018).

### 2.4. qPCR-based bacterial fecal indicator measurements

#### 2.4.1. Water filtration and DNA extraction

Water filtration for qPCR testing was conducted on the same composites used for bacterial and viral fecal indicator culture measurements as described in EPA Method 1611. Briefly, 100 mL of each composite water sample was filtered through a 47 mm diameter, 0.40 μm pore size polycarbonate filter (Millipore, Burlington MA) and stored at − 80 °C. Next, AE buffer (Qiagen, Germantown, MD) containing 0.2 μg/mL salmon testes DNA (Sigma-Aldrich), was added to each sample and subjected to bead milling in an eight-place beater (Biospec Products, Inc., Bartlesville, OK) at the maximum rate for 1 min. DNA was recovered in the supernatant by centrifugation and the clarified supernatant was directly used as a template.

#### 2.4.2. qPCR analysis

Analyses for enterococci, *E. coli* and total *Bacteroidales* gene sequences were performed using previously described assays: Entero1a (USEPA, 2012a, 2012b, 2015a, 2015b); EC23S857 (Chern et al., 2011); and GenBac3 (USEPA, 2010c), respectively. All assays were multiplexed with an internal amplification control (IAC) assay. Target sequences were amplified in 25 μL reactions containing 5 μL of sample DNA extract, 1 μM of each forward and reverse primer, 80 nM each of the FIB target sequence and IAC probes, 5 μg of bovine serum albumin (Sigma-Aldrich), 1X TaqMan Environmental Master Mix (Thermo Fisher Scientific, Microbiology Division, Lenexa, KS) and ~100 copies per reaction of the multi-target IAC plasmid DNA control template (USEPA, 2013a, 2015a, 2015b). The salmon testes DNA spiked into extracts was amplified using the Sketa22 assay (USEPA, 2012a) as described above to evaluate DNA recovery and monitor for PCR amplification interference. All reactions were run in a StepOnePlus™ Real-Time PCR System (Applied Biosystems, Foster City, CA) with initial denaturation at 95 °C for 10 min followed by 40 cycles of 95 °C denaturation for 15 s and 60 °C annealing for 1 min, except for the EC23S857/IAC and corresponding Sketa22 reactions which were annealed at 56 °C. Fluorescence thresholds were set at 0.03 ΔRN and baseline cycles were determined using the software’s AUTO feature.

Blank filter samples (negative controls) containing no target organisms (i.e., negative controls) were prepared in triplicate with each batch of test sample and analyzed in the same manner as test samples. Whole cell calibrators (positive control samples) were also prepared in triplicate with each sample batch and identically analyzed. Enterococci and total *Bacteroidales* gene copies in the test samples were quantified as calibrator sequence equivalents (CSEs) using the Δ-Δ comparative Ct method (USEPA, 2015a, 2015b). The workbook is available (Lane et al., 2020) with the following modification: median estimates of target sequences recovered from the calibrator samples were determined in advance using a weighted linear regression standard curve model and the composited Ct measurement data of plasmid DNA standards from multiple instrument runs as described in Lane et al. (2020). Cultured *Bacteroides thetaiotaomicron* (ATCC #29741) and *Enterococcus faecalis* (ATCC # 29212) cells were used to prepare the calibrator samples as described in EPA Method B and EPA Methods 1611, 1609, 1611.1 and 1609.1, respectively. Calibrator samples also contained *E. coli* (NCTC #12923) cells but were used only as positive controls for the *E. coli* method. *E. coli* gene copies in the test samples were directly quantified using the same standard curve model with composited Ct measurement data generated from analyses of the same multi-target plasmid DNA standards used for the Entero1a and GenBac3 assays (Sivaganesan et al., 2018) using a prototype of the automated Excel analysis workbook presented by Lane et al. (2020). Delta Ct adjustments from the Sketa22 assay were further used in the *E. coli* workbook to adjust for DNA recovery in the test sample extracts as described by Aw et al. (2019). Enterococci CSE estimates were converted in the Method 1611.1/1609.1 workbook to calibrator cell equivalents (CCE) for comparisons with published EPA BAVs (Haugland et al., 2014; USEPA, 2015a, 2015b). All test sample results used in this study were reported per 100 mL of water sample, with the log_10_ copy/reaction quantitative estimates generated by the *E. coli* workbook scaled accordingly.

#### 2.4.3. Data acceptance metrics

Each of the Excel data analysis workbooks referenced above performed automatic checks on standard curves, positive and negative controls, and test sample data quality, and for unacceptable matrix interference in test samples. In the enterococcus and total *Bacteroidales* method workbooks, these checks included: (1) An analysis of covariance (ANCOVA) with an acceptance criterion of *p >* 0.05 for slopes and intercepts of the individual standard curves contributing to the composite curve; (2) target organism and Sketa22 assay Ct measurements for each of the calibrator or positive control sample analyses performed in each test sample run within +/− 3 standard deviations of the means determined for these assays in preliminary analyses; (3) Ct values of duplicate Sketa22 assay analyses of test samples within 3 units of the mean from calibrator sample analyses; (4) Ct values of duplicate IAC assay analyses of test samples within 1.5 units of the mean from negative control sample analyses; and (5) average target organism CSE estimates for the negative control (filter blank) samples analyzed in each test sample run *<*lower limit of quantification (LLQ) value of 720 prior to Sketa22 assay delta Ct adjustments. Undetected Ct measurements were assigned values of 40 for purposes of averaging. The LLQ value established for the EPA enterococci method is 568 CSE (USEPA, 2013b), however, the 720 CSE/sample value was selected as the LLQ for this study based on the analyses of plasmid DNA standards with lowest concentration of 6 copies/reaction and 1/120th of total DNA extract volumes analyzed. Similar data acceptance metrics were applied in the *E. coli* method workbook with the following differences: (1) target organism and Sketa22 assay Ct measurements for each of the positive control sample analyses performed in each test sample run within acceptance bounds established from a multiple laboratory evaluation study of the method (Sivaganesan et al., 2019); (2) mean Ct measurements for the negative control (filter blank) samples analyzed in each test sample run *>* 35.09 LLQ Ct estimate established from the composite standard curve in the workbook (corresponding to 679 copies/sample); (3) standard deviation of duplicate EC23S857 Ct measurements for test samples that were *>* LLQ within 1.414 (Sivaganesan et al., 2019).

### 2.5. Covariate data

Ancillary water and site characteristics were measured as potential statistical covariates. These measurements are the same data as presented in Wanjugi et al. (2018). Instrumentation devices and measurement protocols are described in Table S1 of Wanjugi et al. (2018). All measured covariates are routinely collected for statistical modeling of water quality (Francy et al., 2013; USEPA, 2010a). Water parameters included: water temperature (°C), turbidity (NTU), dissolved oxygen (mg/L), conductivity (μmhos/cm), pH, ultraviolet absorbance in the water column at 254 nm (UV_254, 1/m), and dissolved organic carbon (DOC, mg C/L). Site parameters included: wind speed (km/h), wind direction (ø), air temperature (°C), wave height (m), relative humidity (%), cumulative rainfall (24 h, 48 h, and 72 h; mm), photosynthetically active radiation (PAR, mol/m^2^-s), and counts of humans, birds and dogs at the time of sampling. At Edgewater Beach, the discharge from the Cuyahoga River was available from a permanent gauging station at its mouth (USGS site 04208000). For this analysis, wind speed, wind direction and beach orientation angle were converted into alongshore (Wind-A) and onshore/offshore (Wind-O) wind components using sine and cosine functions (Cyterski et al. 2013). A correlation analysis identified multiple highly collinear covariate combinations (*r* ≥ 0.8): UV_254 and DOC (*r* = 0.81), 24 h and 48 h cumulative rainfall (*r* = 0.84), and 48 h and 72 h cumulative rainfall (*r* = 0.89). To minimize collinearity for regression coefficient estimation, UV_254 and 48h rainfall covariates were excluded from further analyses.

### 2.6. Data analyses

#### 2.6.1. Modeling scenarios and data transformations

Regression models were generated for the seven microbial indicators: F+ coliphage, somatic coliphage, cultured *E.coli*, cultured enterococci, qPCR-based *E.coli*, qPCR-based enterococci, and qPCR-based *Bacteroidales*. Prior to any data analyses, cultured FIB and qPCR-based concentrations (C) were log_10_ transformed due to a large dynamic range with these datasets. Coliphage concentrations were transformed as log_10_(C+1); the small constant added to prevent negative log_10_ values as some coliphage concentrations were *<*1.0. A value of ½ the detection limit (coliphage) or LLQ (qPCR) was used for microbial indicator concentrations under the detection limit or LLQ, respectively. All covariate data were standardized (subtracting the mean and dividing by the standard deviation).

#### 2.6.2. Microbial indicator measurement correlations

Pearson correlation coefficients (r) were used to examine the strength of associations between paired microbial indicator measurements by site. To account for potential variability due to occurrence of non-detects in some data sets, correlation analyses were repeated 100 times for each paired measurement combination and an average r was calculated. For each of the 100 coefficients, microbial indicators below the detection limit (coliphage and cultured FIB) or LLQ (qPCR targets) were assigned a unique uniform random number between zero and the respective detection limit/LLQ. Significance of the correlation coefficients was assessed using a t-test (Kendall and Stuart 1973).

#### 2.6.3. Model formulation

All regression models were developed using the “lars” package (version 1.2, Efron et al. ,2004) in R (version 4.1.3, R_Core_Team, 2021). This package implements an iterative fitting technique (LARS-lasso, Tibshirani, 1997), where the linear regression coefficients are manipulated across successive steps, and Cp (Mallows 1973) is tracked. The “optimum” model is determined by the step where Cp is minimized. This implementation is an L_1_ regularization technique, in which it is possible for regression coefficients for specific covariates to shrink to zero, in essence removing them from the model (i.e., there is no evidence that they are useful).

#### 2.6.4. Model predictive performance evaluation

A cross-validation approach was used to assess predictive performance of each site-specific microbial indicator model. For each model, corresponding data were randomly split into ten folds. Each fold was withheld while the other nine folds were used to train a sub-model. Each sub-model was then used to predict microbial indicator measurements in the withheld data fold. Thus, ten sub-models were developed, resulting in a single prediction for every data point, as each data point occurs in a withheld data fold only once. Predicted microbial indicator values from each set of ten sub-models was used to compute a standardized root mean squared error of prediction (SRMSEP):

$$SRMSEP_{m}=\sqrt{\frac{\sum_{i_{m-1}}^{n_{m}}{\left(P_{i_{m}}-O_{i_{m}}\right)}^{2}}{n_{m}}}/\bar{C_{m}}$$

For each model, n_m_ is the number of observations, $P_{i_{m}}$ is the ith prediction, $O_{i_{m}}$ is the ith observed value, and $\bar{C_{m}}$ is the log_10_ average concentration of the modeled microbial metric. Along with a SRMSEP, $R_{\text{PRED}\;}^{2}$ was calculated via a regression using $P_{i_{m}}$ (predicted values) to $O_{i_{m}}$ (observed values) for each model. For each model, SRMSEP and $R_{\text{PRED}\;}^{2}$ values were also standardized by dividing a given model performance metric by the maximum value observed across all models. Overall Performance for each model was then estimated as follows: [standardized $R_{\text{PRED}\;}^{2}$ + (1 – standardized SRMSEP)]. Standardized SRMSEP was subtracted from one because a high value indicates poor performance.

#### 2.6.5. Covariate evaluation

To assess covariate influence, a single LARS-lasso model was generated for each site and response variable combination using all available measurements for each data set. From this model output, only the regression coefficients were used; models were not used to evaluate predictive performance. For each site, regression coefficients for each microbial indicator and covariate combination (7 microbial indicators, 16 covariates) were displayed as heatmaps (“image” function in base R with color gradient created via the “RColorBrewer” package). The overall influence for each covariate was then evaluated for each covariate across all microbial indicator and site combinations (*n* = 21) based on the following metrics: (1) sum of regression coefficients (absolute values used); (2) frequency of non-zero regression coefficients; and (3) Total Score (sum of metrics 1 and 2). In this analysis, the influence of 24 h and 72 h cumulative rainfall were summed and called “Rainfall.” In the same way, the influence of Wind-A and Wind-O were summed, and this summed influence was called “Wind Speed/Direction.”

## 3. Results

### 3.1. FIB qPCR measurements

**Fig. 2.**
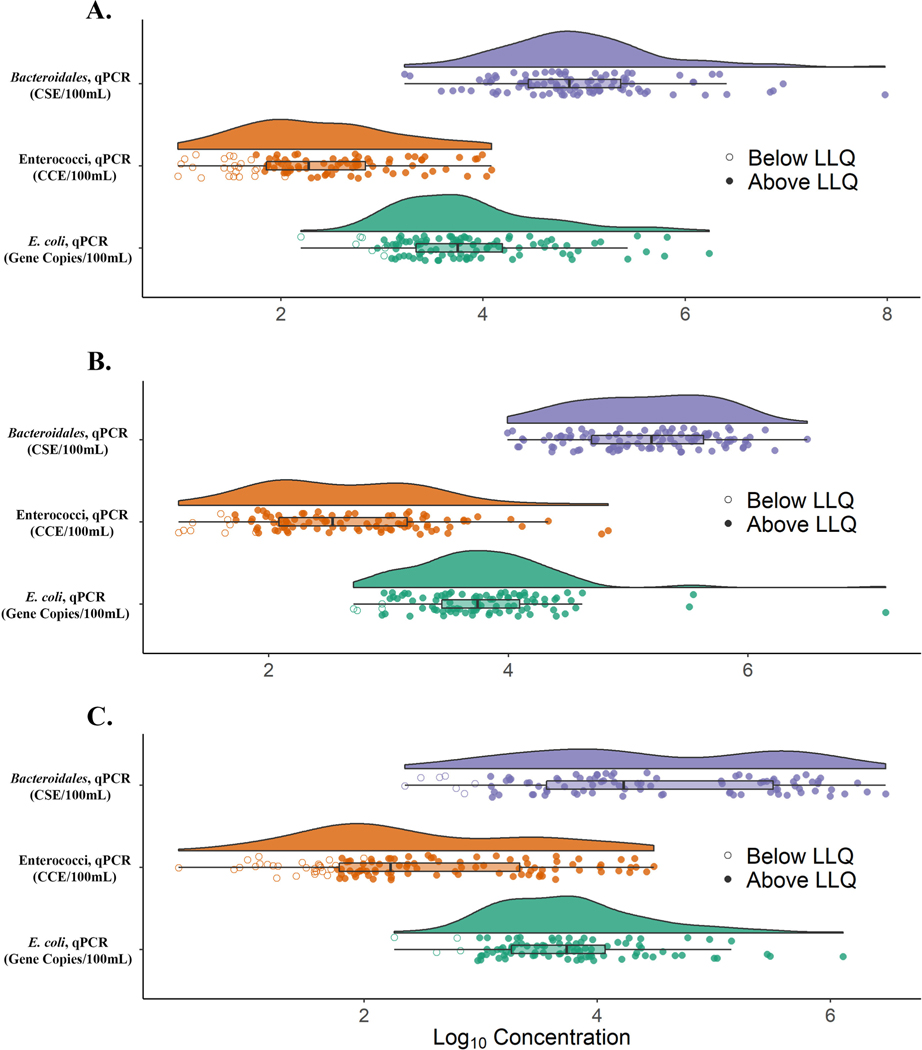
Raincloud plots showing individual qPCR measurements (log_10_ concentration) for Edgewater Beach (Panel A), Grant Park (Panel B), and Washington Park (Panel C) study sites. Open circles show measurements below and shaded circles indicate measurements above the respective detection limit/lower limit of quantification (LLQ). The right/left boundary of each box show the 25th/75th percentiles of the data distribution. The box’s whiskers extend to any observation within 1.5 times the interquartile range. Shaded curves represent respective density distributions.

Overall percentages of water sample measurements that gave *<* LLQ concentration estimates for the Entero1a (enterococci), EC23S857 (*E. coli*), and GenBac3 (*Bacteroidales*) assays were 27.2, 8.1 and 6.2, respectively. Positive and negative control sample acceptance criteria were met in all 50 instrument runs of the test samples for each method from the study (data not shown). Each sample was also evaluated for suitable DNA recovery and absence of amplification interference. These quality controls indicated that the percentage of test sample analyses that failed to meet acceptance criteria in the enterococci*, E. coli* and total *Bacteroidales* methods were 2.25, 2.62 and 3.00, respectively. Composite standard curve performance metrics are summarized in Table 1. Individual qPCR measurements for the three study sites are shown in Fig. 2.

### 3.2. Microbial indicator summary statistics and correlations

**Fig. 3.**
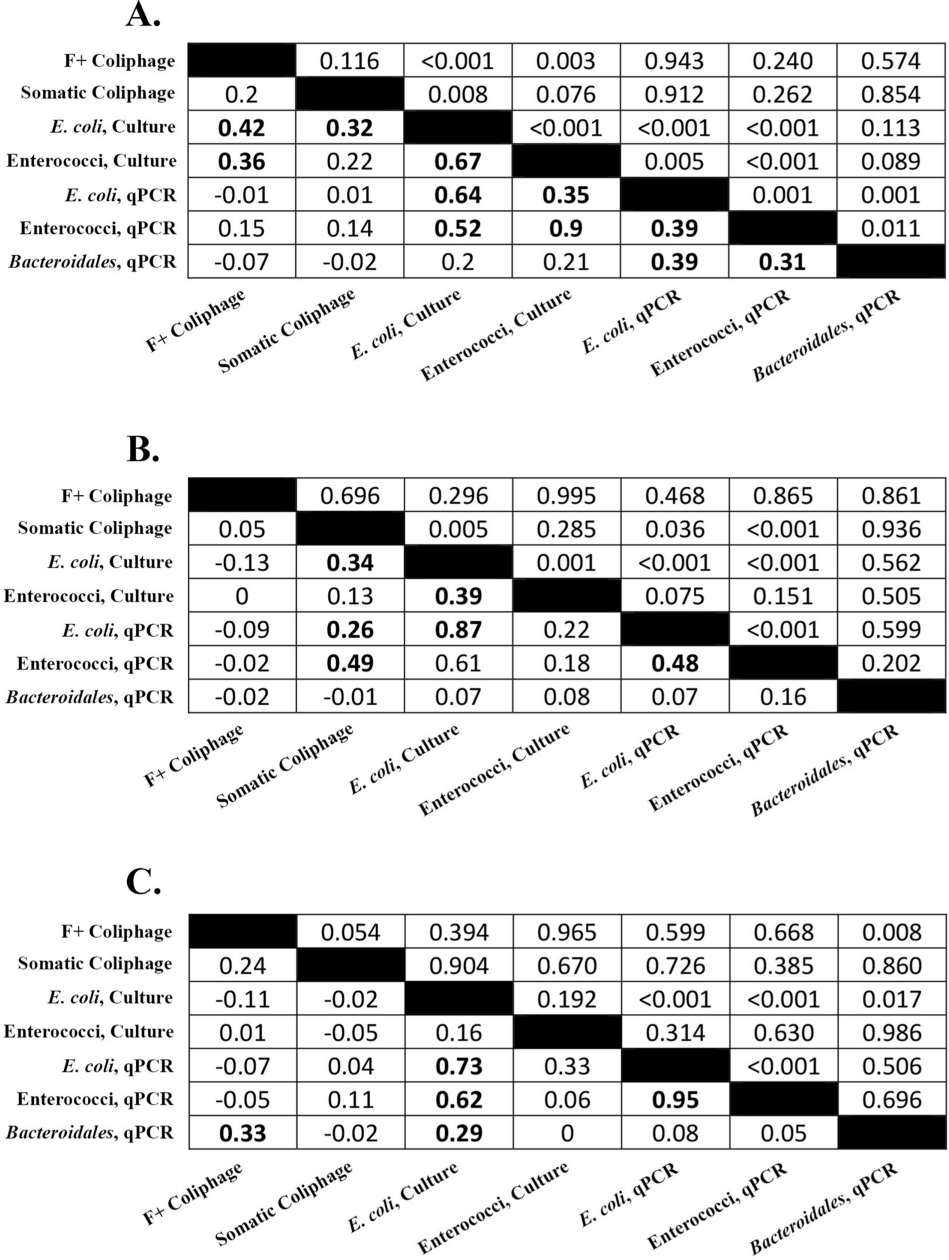
Average Pearson correlation matrices (r) for microbial measures at each study site (A = Edgewater Beach, B = Grant Park, C = Washington Park). Averaged r values appear in the lower left portion of each panel; t-test derived p-values are given in the corresponding upper right cells. Significant coefficients (*p <* 0.05) are bolded. Standard deviations for these coefficients (calculated from 100 simulations where concentrations below detection/LLQ were replaced by random uniform numbers) never exceeded 0.004 and were primarily *<* 0.001.

Microbial indicator measurements targeting two coliphage (somatic and F+), two cultured FIB (enterococci*, E. coli*), and three FIB genetic markers (enterococci, *E. coli*, and *Bacteroidales*) were used as response variables in LARS models. Table 2 shows summary statistics of each data set including: number of total samples, number of sample non-detects and below detection limit (coliphage and cultured FIB) or LLQ (qPCR), number of samples that failed quality controls, and descriptive statistics excluding non-detects [minimum, maximum, mean, standard deviation, and coefficient of variation]. Pearson correlation coefficients (r) between microbial measures varied by site (Fig. 3). Overall, Edgewater Beach showed the most significant correlations (11) of the site-specific datasets, while Washington Park showed the least (5). There were only a few coefficients that were significant at every site: cultured *E. coli* paired with qPCR-based *E. coli*, cultured *E. coli* paired with qPCR-based enterococci, and qPCR-based *E. coli* paired with qPCR-based enterococci.

### 3.3. Predictive Model Performance

**Fig. 4.**
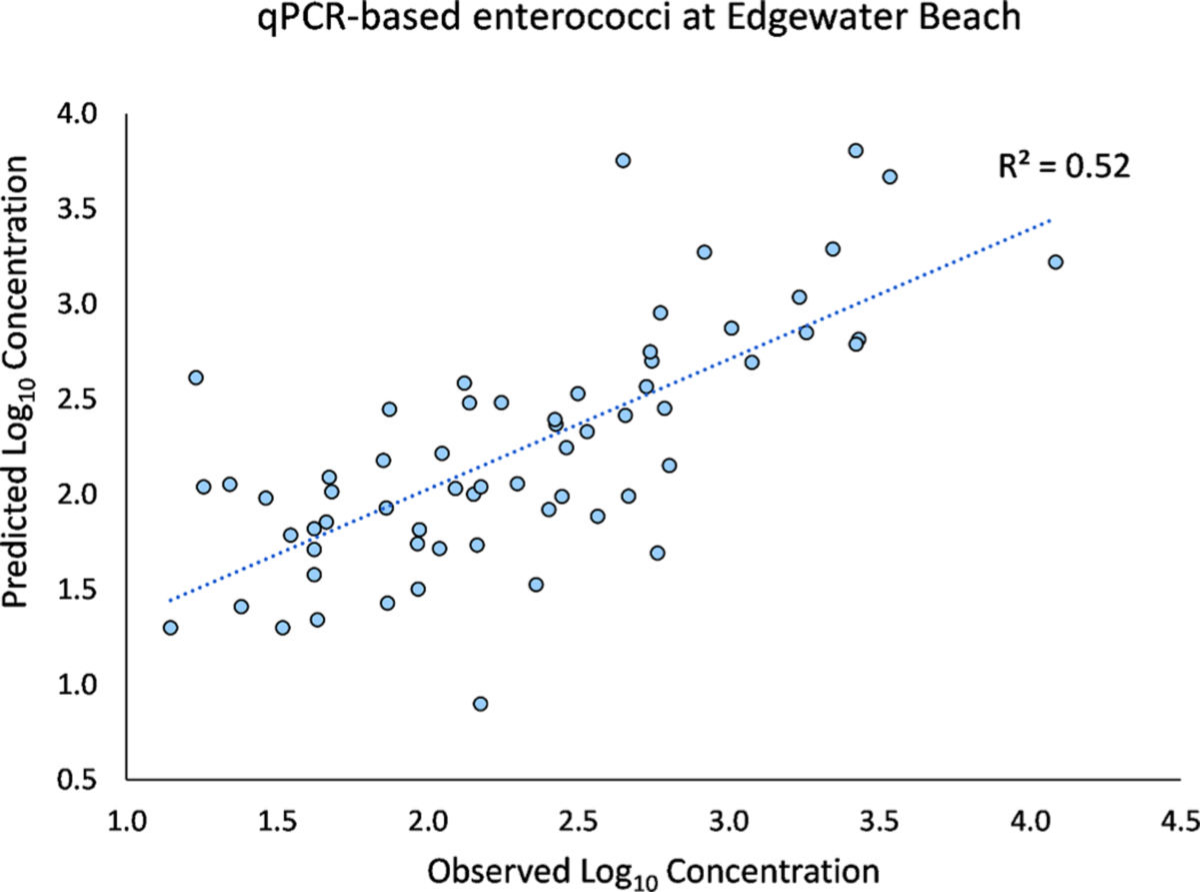
Scatterplot of qPCR-based enterococci (log_10_ CCE/100ml) observed measurements versus LARS sub-model predictions at Edgewater Beach.

Predictive performance metrics for each model (*n* = 21) are given in Table 3. The top performing model (Fig. 4) used the enterococci qPCR Edgewater Beach data set, yielding an $R_{\text{PRED}\;}^{2}$ of 0.52 and SRMSEP of 0.13 (Overall Performance = 1.87). Seventy one percent of Edgewater Beach models (5 of 7) occurred in the top 10 based on the Overall Performance metric. In contrast, 71% (*n* = 5) of Washington Park models yielded Overall Performance scores in the bottom seven, including the poorest performing model (F+, Overall Performance = 0.11). F+ coliphage models exhibited the poorest performance (Overall Performance ≤ 0.31), regardless of site. In addition, all *E. coli* qPCR models resulted in Overall Performance values ranked in the bottom 10, while enterococci qPCR models exhibited the opposite trend (Overall Performance scored ranked in the top 10) including the top performing model. Somatic coliphage model Overall Performance scores ranked 3rd (Edgewater Beach), 7th (Grant Park), and 18th (Washington Park).

### 3.4. Covariate influence

**Fig. 5.**
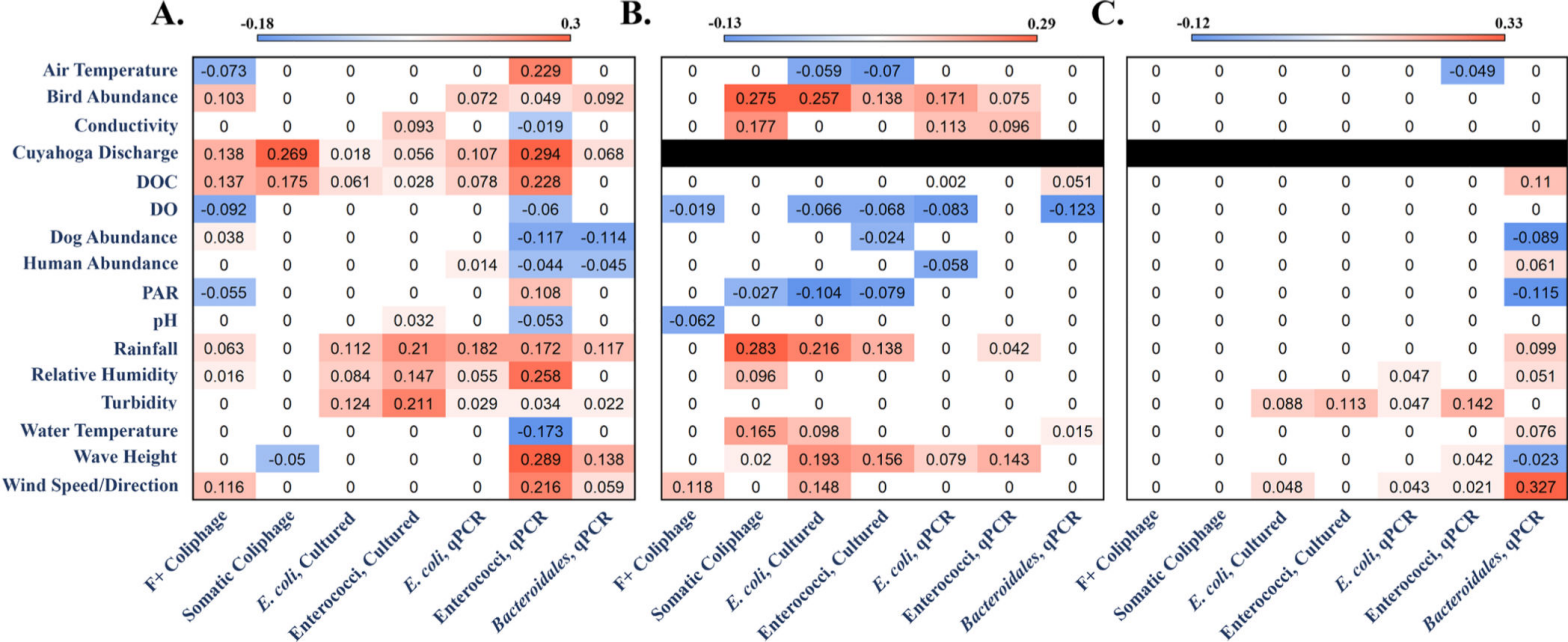
Heat maps of least-angle regression (LARS) regression coefficients for seven microbial indicator predictive models at Edgewater Beach (Panel A), Grant Park (Panel B), and Washington Park (Panel C). Covariates are displayed in alphabetical order on the y-axis and microbial measures are shown on the x-axis. Positive and negative coefficient values are denoted by red and blue shading, respectively. White/pale cells indicate coefficients near or at zero (statistically uninfluential). Cells shaded in black denote no covariate data available for that site.

LARS regression coefficients for each covariate and model combination are shown in Fig. 5. Model covariate regression coefficients ranged from −0.173 (*Bacteroidales* qPCR, Edgewater Beach) to +0.327 (enterococci qPCR, Washington Park). Discharge from the Cuyahoga River at Edgewater Beach was the only covariate with regression coefficients not equal to zero regardless of microbial indicator model (this covariate only available for Edgewater Beach). The somatic and F+ coliphage models at Washington Park were the only instances where all covariate regression coefficients were equal to zero. In contrast, the enterococci qPCR model for Edgewater Beach was the only occurrence where all covariates yielded regression coefficients not equal to zero. The aggregate influence of each covariate across all models is summarized in Table 4. Of the covariates available across all three sites, rainfall exerted the most influence with non-zero regression coefficient values in 52% (11 of 21) of models. Bird abundance, wave height, and wind speed/direction were the next most influential covariates across all microbial indicator models (Total Score ≥ 1.53). Five covariates exerted minimal influence on predictive models (Total Score ≤ 0.74) with pH contributing the lowest (Total Score = 0.29).

## 4. Discussion

### 4.1. Predicting bacterial and viral fecal indicator concentrations in Great Lake recreational waters

This study reports the use of LARS-lasso modeling to predict concentrations of coliphage (F+ and somatic), cultured FIB (*E. coli* and enterococci), and qPCR-based genetic markers (*E. coli*, enterococci and *Bacteroidales*) using water and beach site covariate measurements collected five-days per week over an entire beach season from three Great Lake recreational sites. Due to the limited size of recreational beach season data sets including those reported here, the conventional practice of parsing a dataset into training and testing subsets was not feasible. Instead, a cross-validation approach was used to compare model predictive performance. Findings identified multiple trends. First, a clear difference in predictive model performance was observed between F+ and somatic coliphage types. F+ coliphage models consistently resulted in poor predictive performance, ranking the lowest of all microbial fecal indicators at each recreational beach site. The reduced performance of F+ coliphage models could be due, in part, to a higher incidence of non-detects (25.1% for F+ compared to 2.5% for somatic) and overall lower concentrations in water samples compared to somatic coliphage. As a result, increasing sample volumes could help alleviate this issue. However, working with larger volumes (*>*1 L) presents additional logistical and expense challenges potentially making this solution impractical for routine water quality monitoring. In addition, there is a growing body of evidence suggesting that these virus types exhibit different occurrence patterns in untreated sewage (Korajkic et al., 2020), animal fecal samples (McMinn et al., 2014), and across different surface water types (riverine compared to lake beach) (Wanjugi et al., 2018). In contrast, somatic coliphage predictive modeling exhibited good Overall Performance (Table 3) at Grant Park (1.39) and Edgewater Beach (1.61), suggesting that this viral indicator could be an important fecal indicator tool for future water quality forecasting applications. Second, enterococci and *Bacteroidales* qPCR outperformed *E. coli* qPCR regardless of site. Unlike F+ coliphage, *E. coli* qPCR exhibited a reasonable frequency of non-detects across samples (5.9%) suggesting a different explanation. One possible hypothesis is the presence of naturalized *E. coli* populations in soils and beach sand, a phenomenon that has been reported in multiple Great Lake studies (Ishii et al., 2007; Ishii et al., 2006). The persistence and propagation of naturalized *E. coli* populations could obscure any links between measured covariates and the occurrence of this fecal indicator. Additional research is warranted to investigate the mechanisms resulting in poor performance of F+ coliphage and *E. coli* qPCR fecal indicators.

### 4.2. Microbial measurement correlation trends

New qPCR findings reported here for *E. coli*, enterococci, and *Bacteroidales* add to the previously published data set consisting of paired measurements of cultured FIB and coliphage, providing the opportunity to evaluate correlation trends between seven different microbial fecal indicator water quality metrics. Results are particularly useful because all measurements were generated from the same water sample grabs in the same laboratory utilizing standardized protocols for each methodology. Correlation analyses identified several trends providing potential insights into the co-occurrence or lack thereof between these water quality microbial measures. Average correlation coefficients (r) between cultured *E. coli* and qPCR measurements ranged from 0.64 (Edgewater Beach) to 0.87 (Grant Park) and were significant (*p <* 0.05) regardless of sampling site. In contrast, a significant correlation between enterococci measurements was only observed at one site (Edgewater Beach, *r* = 0.90, *p <* 0.001). Previous studies investigating FIB culture and qPCR paired measurements at Great Lakes beach sites report similar findings, suggesting that the degree of correlation is likely influenced by site specific conditions (Lavender and Kinzelman 2009, Shrestha and Dorevitch 2019, Whitman et al. 2010). While correlations between culture and qPCR measurements of *E. coli* and enterococci are well documented, little is known regarding the level of agreement between coliphage and qPCR FIB metrics. In this study, coliphage exhibited markedly lower correlations with paired qPCR fecal indicator measurements, with most comparisons resulting in non-significant results (p *>* 0.05), suggesting that the occurrence of these viral and bacterial fecal indicators in Great Lake recreational beach waters are governed by different conditions. Potential factors might include different animal source shedding patterns or variable fate and transport behaviors. A recent study comparing coliphage with *E. coli* qPCR paired measurements in untreated sewage samples collected across the contiguous United States reported a similar trend, where somatic coliphage did not significantly correlate with *E. coli* qPCR (*r* = 0.21; *p* = 0.15), but F+ coliphage exhibited a weak correlation (*r* = 0.41, *p* = 0.003) (Korajkic et al. 2020). Further research could help elucidate factors contributing to these different occurrence patterns.

### 4.3. The influence of predictive model covariates

Many covariates were used to predict bacterial and viral fecal indicator concentrations, resulting in several notable trends. Of the covariates used in all models, rainfall was the most influential (Table 4), echoing findings from other microbial water quality modeling efforts focused on cultured FIB at recreational beach sites (Nevers and Whitman, 2005; Whitman and Nevers, 2008). Findings also lend support to the development of a potential core set of physical and beach parameter measurements for future water quality forecasting applications in the Great Lakes basin, as approximately 25% of the covariate data sets exerted minimal influence on microbial indicator predictive models (Table 4). Reducing the number of covariate measurements needed to predict water quality would streamline future applications as well as lower data collection costs. The analysis of covariate influence also provided useful clues about potential fecal sources of pollution at sites. For example, bird abundance was the second most influential covariate across all models (Table 4, Fig. 5), notably at Grant Park and Edgewater Beach. Information on potential sources of fecal pollution can help managers identify sites for future microbial source tracking analyses. Findings also demonstrated the utility of measuring discharge in nearby lotic hydrologic elements (e.g., the importance of Cuyahoga discharge at Edgewater Beach). Both Grant Park and Washington Park are also likely influenced by nearby lotic inputs – Oak Creek and Trail Creek, respectively – but discharge in these systems was not measured in this study due to the absence of permanent gauge stations. The absence of this potentially useful covariate could, in part, have contributed to lower model predictive performance at these two sites versus Edgewater Beach. Additional research is needed to confirm covariate importance trends identified in this study.

## 5. Conclusion

LARS-lasso predictive modeling with cross-validation was used to compare the predictive performance of models of coliphage (F+ and somatic), cultured *E. coli* and enterococci, and qPCR-based *E. coli,* enterococci, and *Bacteroidales* measures using a suite of environmental covariates at three recreational beach sites in the Great Lakes basin. Key findings include:

- Models yielded highly variable SRMSEP and $R_{\text{PRED}\;}^{2}$ measures, indicating that some microbial measures may be more amenable to statistical modeling approaches than others.
- Somatic coliphage models performed at a similar level or better compared to cultured and qPCR FIB while F+ coliphage models consistently performed poorly.
- Enterococci and *Bacteroidales* qPCR outperformed *E. coli* qPCR regardless of beach site.
- Rainfall, bird abundance, wave height, and wind speed/direction were the most influential covariates across all models.
- Approximately 25% of covariates exerted minimal influence on predictive models suggesting a potential core set of physical and beach parameters may be optimal for future water quality forecasting applications in the Great Lakes basin.

Additional research is warranted to further characterize the suitability of statistical predictive models for recreational water quality forecasting of virus and bacterial fecal indicators and confirm trends observed in this study. Findings also provided useful insights on water quality forecasting in the Great Lakes demonstrating a challenging reality, there can be a large degree of variability from one site to another. While LARS allowed for the successful comparison of different microbial indicator predictive performance and identified important differences between coliphage types and qPCR-based fecal indicators, future studies with larger sample sizes could be amendable to alternative approaches such as machine learning techniques that could further improve water quality forecasting.

## Figures and Tables

**Table 1:** Composite qPCR standard curve performance metrics.

Method	Standard Curve		Amplification Efficiency*
		
	*Slope*	*Intercept*	
Entero1a	− 3.49	37.93	0.93
EC23S857	− 3.58	37.78	0.90
GenBac3	− 3.54	37.88	0.92

* Amplification efficiency = [10^(−1/slope)^− 1]

**Table 2:** Summary statistics for microbial indicator log_10_ concentrations measured at the three beach sites. ND = non-detect. Units of measurement for each response and detection limits/lower limit of quantification (LLQ) are given below the table.

	Coliphage		Cultured		qPCR		
			
Edgewater Beach	F+	Somatic	E. coli	Enterococci	E. coli	Enterococci	Bacteroidales
Total Measurements	64	64	66	66	67	67	67
Non-Detect/Below LLQ	15	0	0	0	1	15	0
Failed QC**	0	0	0	0	3	3	5
Minimum	ND	0.93	1.20	0.60	ND	ND	3.27
Maximum	2.03	3.83	3.41	3.61	5.26	4.08	5.89
Mean*	0.42	2.30	2.24	1.85	3.84	2.28	4.75
Standard Deviation*	0.62	0.59	0.50	0.59	0.51	0.68	0.47
Coefficient of Variation	1.50	0.26	0.22	0.32	0.13	0.30	0.10

Grant Park	F+	Somatic	E. coli	Enterococci	E. coli	Enterococci	Bacteroidales

Total Measurements	69	69	71	71	68	68	68
Non-Detect/Below LLQ	16	1	0	0	5	9	0
Failed QC**	0	0	0	0	2	2	2
Minimum	ND	ND	1.00	0.30	ND	ND	3.99
Maximum	1.38	3.08	3.23	3.79	4.84	3.47	6.25
Mean*	0.26	1.66	1.71	1.55	3.56	2.27	4.87
Standard Deviation*	0.51	0.61	0.65	0.70	0.53	0.53	0.47
Coefficient of Variation	1.94	0.37	0.38	0.45	0.15	0.23	0.10

Washington Park	F+	Somatic	E. coli	Enterococci	E. coli	Enterococci	Bacteroidales

Total Measurements	62	62	64	64	67	67	67
Non-Detect/Below LLQ	18	3	0	0	6	25	7
Failed QC**	0	0	0	0	1	0	0
Minimum	ND	ND	0.79	1.00	ND	1.38	2.56
Maximum	1.50	3.06	3.32	4.05	4.73	3.65	5.07
Mean*	0.23	1.63	1.81	1.92	3.34	1.89	3.73
Standard Deviation*	0.49	0.73	0.48	0.59	0.42	0.52	0.60
Coefficient of Variation	2.17	0.45	0.26	0.31	0.13	0.28	0.16
Units	PFU/L	PFU/L	MPN/100mL	CFU/100mL	Gene Copies/100mL	CCE/100mL	CSE/100mL
Detection Limit/LLQ	− 0.097	− 0.097	0	0	2.83	1.68	2.86

* non-detections were set to ½ the detection limit (shown at the bottom of the table) for calculations.

** presumptive sample matrix interference.

**Table 3:** Predictive performance metrics for each microbial indicator and site model.

Site	Microbial Indicator	$R_{\text{PRED}\;}^{2}$	Standardized $R_{\text{PRED}\;}^{2}$	SRMSEP*	Standardized SRMSEP	Overall Performance
Edgewater Beach	F+ Coliphage	0.01	0.02	1.19	0.71	0.31
Grant Park		0.01	0.02	1.50	0.90	0.12
Washington Park		0.06	0.11	1.67	1.00	0.11
Edgewater Beach	Somatic Coliphage	0.38	0.74	0.20	0.12	1.61
Grant Park		0.30	0.57	0.30	0.18	1.39
Washington Park		0.06	0.11	0.45	0.27	0.84
Edgewater Beach	Cultured *E.coli*	0.20	0.38	0.14	0.09	1.29
Grant Park		0.31	0.59	0.22	0.13	1.46
Washington Park		0.02	0.03	0.17	0.10	0.93
Edgewater Beach	Cultured enterococci	0.40	0.76	0.15	0.09	1.67
Grant Park		0.26	0.50	0.23	0.14	1.37
Washington Park		0.02	0.03	0.20	0.12	0.91
Edgewater Beach	*E.coli* qPCR	0.11	0.21	0.13	0.08	1.13
Grant Park		0.07	0.14	0.14	0.09	1.05
Washington Park		0.01	0.01	0.13	0.08	0.93
Edgewater Beach	Enterococci qPCR	0.52	1.00	0.22	0.13	1.87
Grant Park		0.23	0.43	0.21	0.12	1.31
Washington Park		0.18	0.34	0.27	0.16	1.18
Edgewater Beach	*Bacteroidales* qPCR	0.25	0.47	0.09	0.05	1.42
Grant Park		0.09	0.16	0.09	0.06	1.11
Washington Park		0.27	0.53	0.14	0.08	1.44

* SRMSEP denotes standardized root mean squared error of prediction.

**Table 4:** The influence of each covariate across all models.

Covariate	Summed Coefficients	Proportion Occurrence	Total Score
Rainfall	1.63	0.52	2.16
Cuyahoga Discharge*	0.95	1.00	1.95
Bird Abundance	1.23	0.43	1.66
Wave Height	1.13	0.48	1.61
Wind Speed/Direction	1.10	0.43	1.53
Dissolved Organic Carbon	0.87	0.43	1.30
Turbidity	0.81	0.43	1.24
Relative Humidity	0.75	0.38	1.13
Dissolved Oxygen	0.51	0.33	0.85
PAR	0.49	0.29	0.77
Water Temperature	0.53	0.24	0.76
Conductivity	0.50	0.24	0.74
Air Temperature	0.48	0.24	0.72
Dog Abundance	0.38	0.24	0.62
Human Abundance	0.22	0.24	0.46
pH	0.15	0.14	0.29

Summed Coefficients are the sum of the absolute values of LARS regression coefficients in each model.

Proportion Occurrence denotes the proportion of 21 models where a regression coefficient ≠ 0.

Total Score is the sum of ‘summed coefficients’ and ‘proportion occurrence’.

* Values calculated from seven models, not 21, as this covariate was only measured at Edgewater Beach.

## Data Availability

Data will be made available on request.
